# Paraneoplastic disseminated lentigines heralding aggressive Langerhans cell sarcoma

**DOI:** 10.1007/s00277-012-1577-0

**Published:** 2012-09-20

**Authors:** Wing Y. Au, Chris Lai, Nigel J. Trendell-Smith, Wai-Man Ng, Donna L. S. N. Chow

**Affiliations:** 1 Blood-Med Hematology Center, Hong Kong, People’s Republic of China; 2 Department of Pathology, Baptist Hospital, Hong Kong, People’s Republic of China; 3 Department of Pathology, Queen Mary Hospital, Hong Kong, People’s Republic of China; 4 Pedder Surgical Group, Hong Kong, People’s Republic of China; 5 Hong Kong Pacific Cancer Centre, Hong Kong, People’s Republic of China; 6 1108 Crawford House, 70 Queen’s Road Central, Hong Kong, People’s Republic of China

**Fig. 1:**
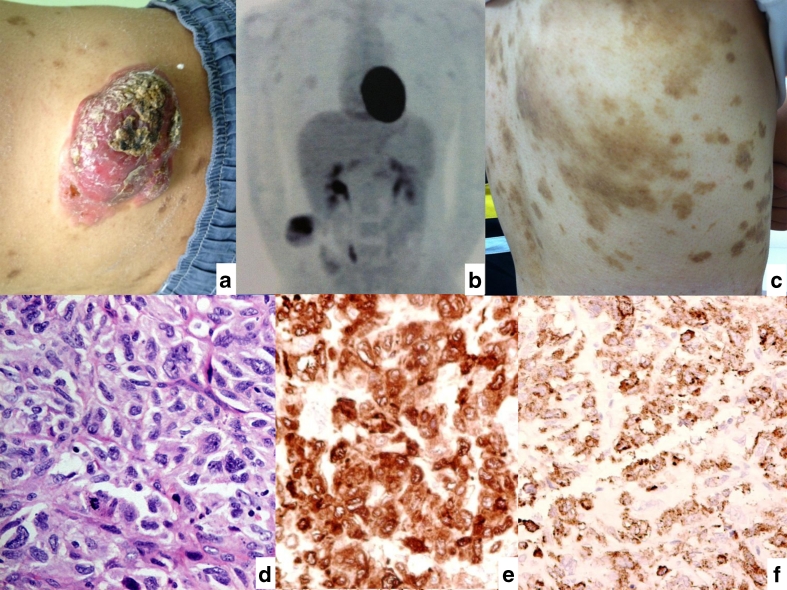
**a** Clinical photo showing the flank mass with impending ulceration. **b** Widespread lentiginous lesion all over the trunk heralding the rapid growth of the malignant lesion. **c** PET with maximum intensity projection showing two sites of active lesions in the flank and in the right groin lymph node. **d** Histology of the lesion showing sheets of pleomorphic tumor cells with indented or lobulated nuclei, moderate to severe nuclear pleomorphism, distinct nucleoli, and moderate pale eosinophilic or amphophilic cytoplasm. Occasional multinucleated cells are present, and mitotic count is frequent. (H&E, ×400 magnification). **e** The tumor cells are positive for CD1a stain. (Immunotech 1:20, ×400 magnification). **f** The tumor cells are positive for Langerin stain with cytoplasmic granular staining. (Novocastra 1:200, ×400 magnification)

Dear Editor,

A 21-year-old man presented with a rapidly growing (9 cm), non-tender right flank mass with superficial excoriation and inflammation (Fig. 1a). The lesion was accompanied by rapid onset of disseminated brown papules, involving the torso, back, and limbs (Fig. 1b). Facial and axillary skins were spared, and there was no particular direction of spread. The skin patches did not coalesce or show pruritus or inflammation. There was also weight loss of 15 lb in 3 months. A positron emission tomogram scan (PET) showed FDG uptake in the right waist (SUV 5.8) and right groin (SUV 2.1) (Fig. 1c). Wide margin resection of the mass and lymph node excision was performed with clear margins. Histology of both specimens showed sheets and loose clusters of pleomorphic malignant cells infiltrating the dermis and subcutis with frequent mitosis (Fig. 1d). In view of the anaplastic morphology and unusual presentation, extensive immunophenotyping was performed. The cells were positive for CD1a, S100, and Langerin stains. They were negative for melanocytic markers (HMB45, melan-A, MITF) EMA, cytokeratin (CK, Cam 5.2, 34betaE12), actin, desmin, CD31, CD34, c-kit, B and T markers (CD20, CD79a, CD3), CD68, CD30, CD56, CD21, MPO, and ALK1. Electron microscopy for Birbeck granules was not performed. The picture was compatible with Langerhans cell sarcoma (LCS). A separate skin biopsy of a pigmented lesion on the torso showed elongated rete ridges with increased basal melanin pigmentation within keratinocytes and the tips, with no increased Langerhans cells and no evidence of malignancy or dysplasia. The lesion was compatible with common lentigo. The patient was treated with consolidation radiotherapy to the primary sites, but there was no regression of the skin lentiges.

In the World Health Organization classification, LCS is the malignant end of the spectrum of benign Langerhans cell histiocytosis (LCH) [1]. The typical LCH cell is replaced by pleomorphic tumor cells in the LCS, only identifiable by phenotype or ultrastructure. Initial unifocal subcutaneous involvement is not unusual. However progressive disease, especially with multifocal dissemination, is highly fatal, despite multi-agent chemotherapy. It remains to be seen whether systemic progression may occur in our patient.

Our patient demonstrated two hitherto unreported phenomena, namely paraneoplastic manifestation of LCS and acquired rampant disseminated lentigines as a new paraneoplastic dermatosis. The clinical history and histological findings do not suggest an underlying disseminated cutaneous LCH. Common paraneoplastic dermatoses (e.g., acanthocis nigricans) are well characterized and are putatively caused by neoplastic humoral secretions. Acquired melanocytic nevi are mostly related to solar exposure and are therefore localized. Recently, some cases of acral lentigines are debated to be paraneoplastic [2]. Disseminated lentigines are usually only found in familial lentiginosis syndromes (e.g., LEOPARD syndrome, Peutz–Jeghers, Carney complex, PTEN hamartoma syndrome). The neoplastic associations of these syndromes come from their primary systemic gene mutations [3]. The rapidly acquired disseminated growth of lentigines in our case is highly unusual and is somewhat induced by the abnormal aggressive transformation of the LCS cells. Skin pigmentation is usually under exquisite humoral control [4], and cutaneous Langerhans cells interact closely with melanocytes and keratinocytes [5]. It is impossible, however, to retrospectively work out the exact mechanism how the localized growth induced a systemic pigment response, since interval archival specimens are unavailable and the list of possible mediators are extensive. In any case, extirpation of the primary lesion appeared to halt further skin lesions.
